# Altered Synaptic Plasticity in Tourette's Syndrome and Its Relationship to Motor Skill Learning

**DOI:** 10.1371/journal.pone.0098417

**Published:** 2014-05-30

**Authors:** Valerie Cathérine Brandt, Eva Niessen, Christos Ganos, Ursula Kahl, Tobias Bäumer, Alexander Münchau

**Affiliations:** 1 Institute of Neurogenetics, University of Lübeck, Lübeck, Schleswig-Holstein, Germany; 2 Department of Neurology, University Clinic Hamburg-Eppendorf, Hamburg, Hamburg, Germany; 3 Institute of Neuroscience & Medicine, Research Centre Jülich, Jülich, Nordrhein-Westfalen, Germany; 4 Sobell Department of Motor Neuroscience and Movement Disorders, University College London, London, United Kingdom; Università di Trento, Italy

## Abstract

Gilles de la Tourette syndrome is a neuropsychiatric disorder characterized by motor and phonic tics that can be considered motor responses to preceding inner urges. It has been shown that Tourette patients have inferior performance in some motor learning tasks and reduced synaptic plasticity induced by transcranial magnetic stimulation. However, it has not been investigated whether altered synaptic plasticity is directly linked to impaired motor skill acquisition in Tourette patients. In this study, cortical plasticity was assessed by measuring motor-evoked potentials before and after paired associative stimulation in 14 Tourette patients (13 male; age 18–39) and 15 healthy controls (12 male; age 18–33). Tic and urge severity were assessed using the Yale Global Tic Severity Scale and the Premonitory Urges for Tics Scale. Motor learning was assessed 45 minutes after inducing synaptic plasticity and 9 months later, using the rotary pursuit task. On average, long-term potentiation-like effects in response to the paired associative stimulation were present in healthy controls but not in patients. In Tourette patients, long-term potentiation-like effects were associated with more and long-term depression-like effects with less severe urges and tics. While motor learning did not differ between patients and healthy controls 45 minutes after inducing synaptic plasticity, the learning curve of the healthy controls started at a significantly higher level than the Tourette patients' 9 months later. Induced synaptic plasticity correlated positively with motor skills in healthy controls 9 months later. The present study confirms previously found long-term improvement in motor performance after paired associative stimulation in healthy controls but not in Tourette patients. Tourette patients did not show long-term potentiation in response to PAS and also showed reduced levels of motor skill consolidation after 9 months compared to healthy controls. Moreover, synaptic plasticity appears to be related to symptom severity.

## Introduction

Gilles de la Tourette syndrome (GTS) is a neuropsychiatric disorder characterized by motor and phonic tics that typically emerge during early childhood [1]. Tics are patterned repetitive movements resembling voluntary movements but are misplaced in context and time [2]. They are often preceded by inner urges to move, which have been associated with abnormal activation in the supplementary motor area (SMA) [3]. It has been repeatedly shown that GTS patients have inferior performance in some motor learning tasks [4], [5] as well as reduced synaptic plasticity as induced by transcranial magnetic stimulation (TMS) [6], [7]. However, to our knowledge, it has not been investigated whether altered synaptic plasticity is directly linked to impaired motor skill acquisition in GTS patients.

Synaptic plasticity refers to the capacity of nerve cells to alter their structural and functional properties such as strengthening of a synapse by long-term potentiation (LTP). LTP is defined as an activity dependent long lasting enhancement of synaptic transmission, while long-term depression (LTD) refers to a long lasting attenuation of synaptic transmission; both LTP and LTD are referred to as synaptic plasticity and are thought to constitute the neuronal basis for learning and memory [8], [9]. Synaptic plasticity can be induced via temporally correlated pre- and post-synaptic activation. The relative timing of this activation determines whether the synapse is strengthened or weakened. In many neuronal systems LTP occurs if the presynaptic neuron fires in a critical interval prior to the post-synaptic neuron and LTD occurs if the post-synaptic neuron fires prior to the pre-synaptic neuron [10]. Activation dependent plasticity is also called Hebbian learning and is based on an enhanced influx of calcium through *N*-methyl-D-aspartate (NMDA) receptor gated channels [11], [12] or by activation of voltage-dependent calcium channels [13]. This leads to a change in the number of glutamatergic α-amino-3-hydroxy-5-methyl-4- isoxazole propionic acid (AMPA) receptors [14]. Studies in single cells and animals suggest that LTP is expressed via inactive, postsynaptic AMPA receptors diffusing into the synaptic cleft, thereby strengthening synaptic transmission, while LTD is likely expressed by a reduction in postsynaptic AMPA receptors via endocytosis [14]. Accordingly, synaptic plasticity does not occur if NMDA receptors are blocked [10], [11], [15]

LTP – and LTD-like neuroplasticity can be induced in the primary motor cortex (M1) in humans using techniques such as repetitive TMS protocols including theta burst stimulation (TBS), high frequency stimulation (HFS) and paired associative stimulation (PAS). It has been shown that PAS induces synaptic plasticity more effectively than TBS, at least in healthy participants [16].

In the PAS protocol, an electrical, peripheral stimulus is applied to the wrist, before a TMS stimulus is delivered to the contralateral M1. The PAS protocol displays properties that are also associated with synaptic plasticity induced in single cells. First of all, if the peripheral, afferent stimulus arrives at the same time or shortly before the TMS stimulus in M1 (approx. 25 ms interval between the stimuli – PAS_25_), corticospinal excitability increases [17]; if the afferent arrives after the TMS stimulus (approx. 10 ms interval – PAS_10_), excitability decreases [18]. Secondly, the change in excitability is specific to the cortical representation of the stimulated cutaneous region [17]–[19]. Thirdly, both LTP-like and LTD-like plasticity is likely mediated by synapses of excitatory neurons [20], [21] and cannot be induced if NMDA receptors are blocked [18], [22]. Instead of using a default 25 ms interval for the excitatory PAS protocol, the interstimulus interval can also be determined on an individual basis by measuring how long an electrical stimulus takes to travel form the wrist to the cortex (N20 latency - PAS_N20_).

Motor evoked potentials (MEP) are commonly measured as the dependent variable in TMS paradigms inducing synaptic plasticity because they reflect corticospinal excitability. Studies in healthy participants show an increase in MEP amplitudes after PAS_25_ and a decrease after PAS_10_ [17], [18], [23]. It has to be kept in mind though that altered MEP amplitudes at the same stimulus intensity could be the consequence of changes in the synaptic weights at excitatory neurons or inhibitory neurons. However, it has been repeatedly been shown that the effects of PAS on inhibitory synapses is not strong enough to induce long lasting excitability changes [20], [24], [25].

Previous studies employing TBS and HFS have found reduced synaptic plasticity in GTS patients as compared to healthy controls [6], [7]. These findings have important implications for understanding which neural processes may cause GTS patients to experience difficulties in motor learning but have to be regarded with some care because both studies included GTS patients with comorbid obsessive–compulsive disorder (OCD) or attention deficit hyperactivity disorder (ADHD). To our knowledge, there are no published studies investigating the effects of PAS in GTS patients. In addition, several lines of studies indicate that GTS patients display deficits in tasks of visuo-motor integration. However, most studies did not control for comorbid ADHD [26]. Neither children [27]–[29], nor adults [30] with GTS show deficits in simple motor speed tasks but both display deficits in fine motor skill tasks requiring visuo-motor integration [27]–[30]. Although it has already been shown that deficits in fine motor skills in childhood can predict tic severity in adult GTS patients [5], there are no published studies investigating long-term consolidation of motor skills (or lack thereof) in GTS.

In order to relate motor skill acquisition and consolidation to synaptic plasticity in GTS patients, we investigated whether synaptic plasticity in M1 is impaired in uncomplicated GTS patients, employing the adapted PAS protocol as described by Ziemann and colleagues (2004) [31]. Moreover, we investigated how synaptic plasticity in GTS patients and healthy controls is related to short-term and long-term motor learning using the rotary pursuit task. While the execution of the rotary pursuit task engages a wide network of brain regions located in the cortex, the striatum and the cerebellum, motor learning is correlated with increased activity over time in contralateral M1, SMA and pulvinar of the thalamus [32], hence synaptic plasticity in M1 and motor learning in the rotary pursuit task should be associated. Moreover, long-term improvement of motor performance in the rotary pursuit task has been shown to be associated with previously induced LTP in healthy participants [33], and an association between PAS_25_-induced LTP and motor learning in the rotary pursuit task has been further demonstrated in a sample of healthy controls and schizophrenia patients [34].

Resulting effects of PAS_N20_ were determined on the basis of MEP amplitudes and cortico-spinal excitability measured by input-output (IO) curve changes [35]. While MEP changes have mainly been associated with short-term effects of cortical plasticity, changes in IO curves are thought to reflect more long-term changes in cortical plasticity, likely connected to consolidation processes through synaptogenesis [36].

The aim of this study was to corroborate findings of altered synaptic plasticity in uncomplicated GTS patients and relate synaptic plasticity directly to the ability to acquire and consolidate motor skills. Our results confirm that synaptic plasticity was altered in GTS patients as compared to healthy controls. While motor skill acquisition was normal in GTS patients, motor skill consolidation was impaired and only correlated with induced LTP in healthy controls but not in patients.

## Materials and Methods

### Participants

Fourteen patients (mean age of 25.6 years, SD  = 5.9; 13 males) with a diagnosis of GTS according to DSM-IV-TR criteria were recruited from the University hospital Hamburg-Eppendorf in Hamburg. Patients fulfilling criteria for OCD according to the structured clinical interview for DSM-IV Axis I disorders (SCID-I), ADHD according to DSM-IV-TR or other neurological or psychiatric comorbidities were excluded from the study. Thus, all patients had uncomplicated GTS exhibiting no clinically relevant comorbidities. GTS symptom severity within a week before the study was assessed by a clinician using the Yale Global Tic Severity Scale (YGTSS) [37]. Additionally, lifetime likelihood of GTS was assessed using the Diagnostic Confidence Index (DCI) [38] because tics tend to wax and wane. At the time of the study, all patients reported motor tics and an additional 5 reported having vocal tics. Mean disease duration was 19.7+/−6.7 years. Mean DCI score was 47.8+/−7.9, mean YGTSS total tic severity was 15.71+/−5.8, mean YGTSS motor tic severity was 12.42 +/−4.3 and mean YGTSS vocal tic severity was 3.29+/−4.2. Four patients were taking medication at the time of the study (please see table 1). Information about premonitory urges, assessed by the validated German version of the “Premonitory Urge for Tics Scale” (PUTS) [39] was available for 12 patients (M = 23.3+/−4.7). The PUTS was originally developed for children but has recently been validated also in adult GTS patients [40], [41].

**Table 1:** Demographic Data & Clinical Assessment.

	Gender	Age (years)	DCI	YGTSS	YGTSS	YGTSS	Medication
	(M:F)	Mean (SD)		Vocal Tic Severity	Motor Tic Severity	Total Tic Severity	
Healthy controls	13∶1	25.7 (4.4)					0
GTS	12∶3	25.6 (5.9)	47.8	3.29 (4.2)	12.43	15.71	4:
patients			(7.9)		(4.3)	(5.8)	Amisulprid, Tegretol retard, Tiapridex, Tiaprid

Data shown are means and standard deviations (SD) for age, Diagnostic Confidence Index (DCI) and the Yale Global Tic Severity Scale (YGTSS); GTS  =  Gilles de la Tourette syndrome.

Fifteen healthy age-matched individuals (mean age of 25.7 years SD  = 4.4; 12 males) without a history of psychiatric disorders or neurological diseases were recruited as a control group. All participants were tested between 1–7 pm to avoid confounding effects of circadian rhythm. All participants were right-handed as assessed by the Edinburgh handedness Inventory [42] and gave their written informed consent prior to the study. This study, including all measures and interventions, was reviewed and approved by the ethics committee of the “Ärtzekammer Hamburg” and conformed to the Declaration of Helsinki.

Before the start of the experiment, all participants completed a TMS safety screening. None of the participants had a family history of epilepsy or had undergone neurosurgery. Thereafter, the PAS_N20_ protocol was administered. MEPs were measured before PAS_N20_, immediately after PAS_N20_ and 30 min after PAS_N20_. Participants were given a 10 min break and were then asked to complete 12 trials of the rotary pursuit task, overall 45 min after administration of the PAS_N20_ protocol. Additionally, all participants were invited for a second testing session of the rotary pursuit task 9 months later. Of the 29 participants, 10 patients and 12 healthy controls were able to attend the second testing session.

### Experimental procedure

Participants were seated in a comfortable reclining chair with their hands resting on a table and were asked to relax and keep their eyes open. To insure that all participants stayed alert during the whole TMS procedure, a standardized attention test was administered [43]. Participants were instructed to look at the stimulated hand, count light stimuli projected onto this hand during the experiment (produced with a laser pointer) and later report how many stimuli they had counted overall. The number of light stimuli ranged from 5 to 7 in all participants.

The optimal location for the magnetic coil was defined as the site where the largest MEPs in the right abductor pollicis brevis (APB) muscle could be produced by slightly suprathreshold stimulation of the contralateral M1. The location and orientation of the coil was then marked on the scalp with a soft pen. Next, the resting motor threshold was determined as the lowest stimulus intensity capable of inducing peak-to-peak MEPs with amplitudes of more than 50 µV in the relaxed APB in at least 5 out of 10 consecutive trials. TMS stimulus intensities are generally reported as percentage of maximum stimulator output (100%). The test stimulus intensities applied during all following stimulations were adjusted to evoke peak-to-peak MEP amplitudes of approximately 1 mV in each participant. The sensory perception threshold for peripheral stimulation was defined as the least intense electrical stimulus that could be perceived by each participant and was assessed by increasing and decreasing stimulus intensity 10 times around the first noticeable stimulus.

Somatosensory evoked potentials were then obtained from each participant to assess how long it takes for an electrical stimulus to travel from the median nerve at the wrist to the cortex. For this purpose, 300 electrical stimuli (200 µs duration, 3 x sensory perception threshold) were applied to the right median nerve and the average response time was measured over sensory motor cortex (at C3, as the active electrode and Fz, as the reference electrode). For reliability purposes, somatosensory evoked potentials were measured twice and results were averaged. Based on this method, the interval between the electrical stimulus applied to the wrist and the magnetic stimulus applied to the cortex in the PAS protocol can be individualised and thus optimised for each participant.

### Paired-associative stimulation (PAS) protocol

**Figure 1:**
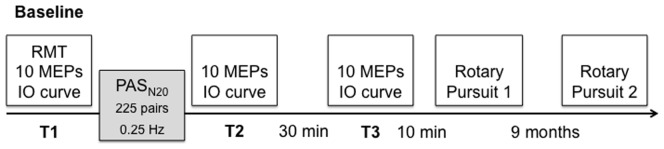
Experimental design. 10 motor evoked potentials (MEPs), and input-output (IO) curves at five different intensities served as dependent variables. They were assessed three times: before the application of the paired associative stimulation (PAS_N20_) paradigm, immediately after PAS_N20_ and 30 minutes later. The rotary pursuit task was carried out following TMS measurements 45 min after PAS_N20_ and 9 months later.

PAS is a conditioning paradigm. Peripheral, electrical stimulation at the wrist and central, TMS stimulation over M1 are repeatedly combined in such a way that both stimuli arrive in the cortex simultaneously, which should result in a transient strengthening of the synapses involved. The PAS_N20_ consisted of 225 pairs of single, peripheral electrical stimuli at the median nerve (300% of the sensory perception threshold) and suprathreshold TMS over the hand area of the contralateral M1 (adapted from [44], [45]). Individual interstimulus intervals between the peripheral and the cortical stimulus were adjusted according to the respective result of the somatosensory evoked potentials. These paired peripheral and cortical stimuli were delivered at 0.25 Hz for 15 min. Peak-to-peak amplitudes of MEPs were measured prior to PAS_N20_ (T1) (average of 10 MEPs, given at a rate of 0.1 Hz with an inter-trial interval variability of 25%.), immediately after PAS_N20_ (T2) and 30 min later (T3) (for the timeline please see figure 1).

IO curves were determined three times subsequent to each MEP measurement. IO curves constitute the relationship between TMS stimuli applied at different intensities and biological responses, and provide additional information about cortico-spinal excitability at different intensities. To this end, MEPs were determined at five different stimulus intensities (100%, 110%, 120%, 130%, 140% of the resting motor threshold). The measured output slope of IO curves is sensitive to the order in which the stimulus intensities are applied (ascending or descending), hence five single pulses per intensity were delivered twice in an ascending order of stimulus intensity.

### Transcranial Magnetic Stimulation

Surface electromyography (EMG) recordings were made with silver surface electrodes placed over the right APB using a tendon-belly montage. EMG signals were amplified and filtered (20 Hz to 1 kHz). The signals were sampled at 5000 Hz and digitized using an analogue-digital converter (Micro1401, Cambridge Electronics Design (CED), Cambridge, UK). Off-line data analysis was performed with the Signal software (Cambridge Electronics Design, Version 3.10). Auditory feedback on muscle relaxation was provided through a loudspeaker connected to the EMG channel.

All TMS measurements were performed with a Magstim 200 magnetic stimulator (Magstim Company, Whitland, Dyfed, UK). A figure-of-eight coil with an outer diameter of 70 mm (Magstim Company) was held tangentially over the scalp at a 45° angle to the sagittal plane with a coil orientation inducing posterior-anterior currents in the brain.

Electrical stimulation for somatosensory evoked potentials measurements and during the administration of the PAS_N20_ protocol was applied over the median nerve at the wrist with a standard stimulation block (cathode proximal) at a stimulation width of 200 µs and a duration of 1 ms.

### Rotary Pursuit Task

The motor skill task consisted of a computerized version of the rotary pursuit task [46]. Participants have to keep a tracking arrow on top of a red dot that moves around on a circle. The dependent measure is “time on target”, i.e. the duration per trial a participant manages to keep the curser of the mouse on the red dot. Each trial lasts 15 seconds. Participants completed 3 blocks, each consisting of 4 trials.

### Data analysis

MEP amplitudes were measured semi-automatically peak-to-peak for each frame using Signal software (customized script). Mean values were calculated for each participant by averaging the MEP amplitudes, excluding single trials that deviated more than 2.5 SDs from the mean. The data pre-processing was also conducted by script. Subsequent off-line data analysis in SPSS was not conducted blind to the diagnosis.

Repeated measures analyses of variance (ANOVA) were carried out with time (T1, T2, T3) as a within-subjects factor and group as a between-subjects factor to detect differences in mean MEP amplitude in response to PAS_N20_. In case of a violation of the sphericity assumption, the Greenhouse-Geisser correction was chosen. Post-hoc tests, if applicable, were conducted using independent samples *t*-tests and paired-samples *t*-tests. Results were considered significant if *p*<0.05. An “MEP change” variable was calculated by subtracting mean MEP amplitude values at T2 from values at T1 so that positive values represent LTP-like changes and negative values represent LTD-like changes. In addition, to determine synaptic plasticity independent of the direction (LTP or LTD), absolute values of MEP changes from time 1 to time 2 (|MEP T2-T1|) were compared between the groups by an independent samples *t*-test.

To evaluate PAS_N20_ effects on IO curves, 3×5 repeated measures ANOVAs with time (T1, T2, T3) and stimulus intensity (100%, 110%, 120%, 130%, 140%) as within-subjects factors were carried out for both groups. Slopes of each curve were assessed for each participant by fitting the data to a linear regression function. The slope values were then entered into a repeated measures ANOVA with time (T1, T2, T3) as a within subjects factor and group as a between-subjects factor. Hypothesis-driven correlations were performed in patients between clinical scores, MEP change, IO curve slopes, resting motor threshold and strength of test stimulus.

To investigate differences in motor learning between the groups, a repeated measures ANOVA was carried out with trial (1–12) and time (rotary pursuit 1, rotary pursuit 2) as within-subjects factors and group as a between subjects factor. Further, MEP change was correlated with motor performance. Two healthy controls had 4 missing data points at rotary pursuit 1. The missing data points were replaced by the mean values of all other healthy controls for the respective trial. For correlation analyses, the 4 trials of the 3 blocks were averaged for time 1 and 2 respectively.

## Results

Healthy controls and GTS patients performed equally well in the attention test administered during PAS_N20_ [*t*(27)  = .21, *p* = .837]. MEPs before and after PAS_N20_ were obtained from 14 GTS patients and 15 healthy controls. IO curve data were not available in one GTS patient because this patient experienced higher stimulation intensities as uncomfortable. Groups did not differ with respect to gender or mean age (see table 1). Also, mean resting motor threshold,strength of test stimulus, somatosensory evoked potentials latencies and sensory perception threshold did not differ significantly between groups (table 2). Age was neither correlated with MEP size at T1 (*r* = .08) nor with MEP change from T1 to T2 (*r* = −.07).

**Table 2:** TMS Parameters.

	Somatosensory evoked potentials (ms)	Resting motor threshold (% stimulator output)	Test stimulus (% stimulator output)	Sensory perception threshold (mA)
Healthy controls	22.4 (1.5)	44.0 (5.8)	56.3 (8.4)	2.4 (1.1)
GTS patients	21.8 (1.2)	47.1 (8.6)	58.1 (11.6)	2.2 (0.6)

Means and standard deviations (SD) of somatosensory evoked potentials, resting motor threshold, test stimulus intensity and the sensory perception threshold during paired associative stimulation (PAS) are shown; GTS  =  Gilles de la Tourette syndrome

**Figure 2:**
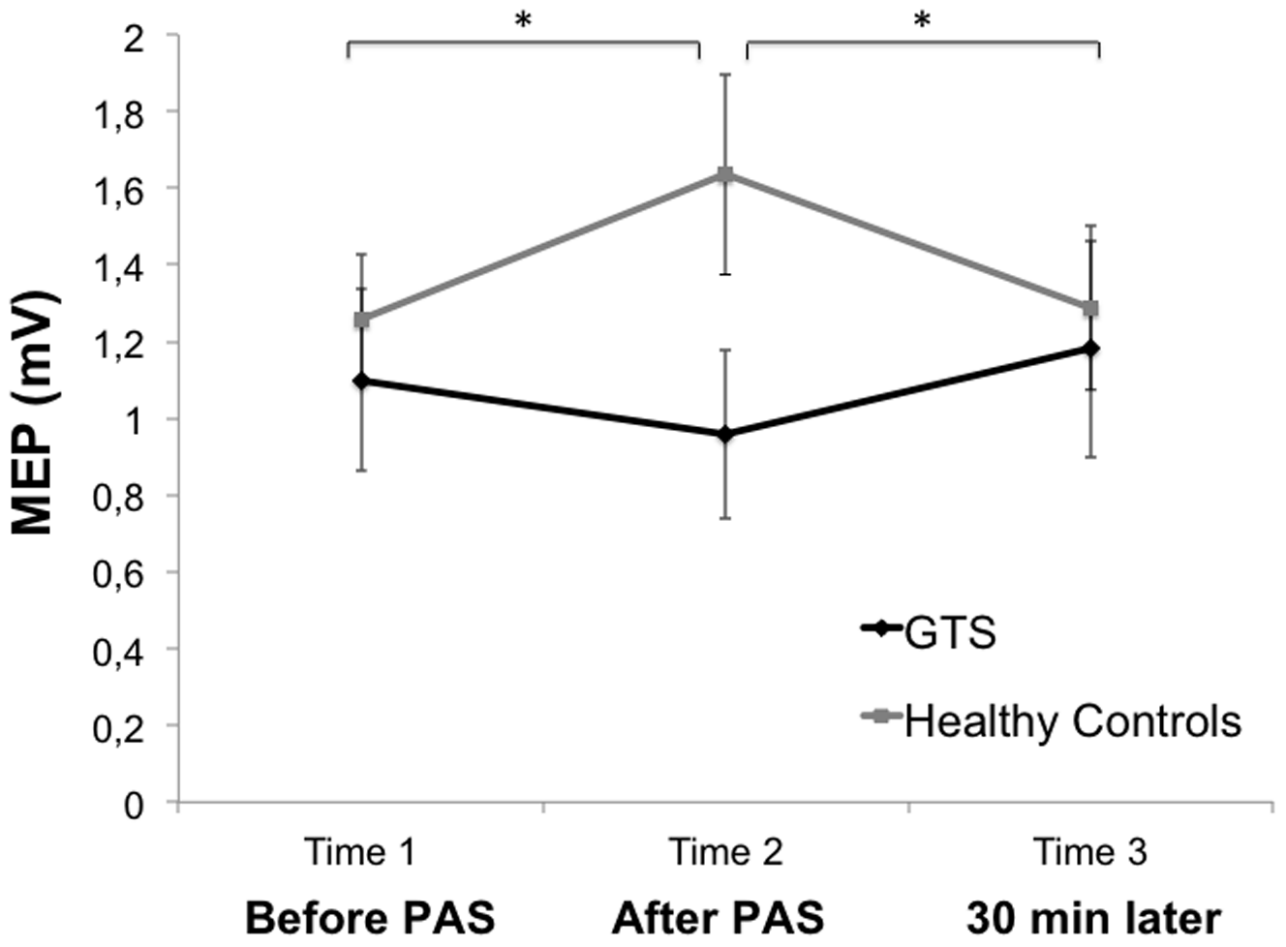
Mean MEP amplitudes at T1, T2 and T3. Data shown are mean values +/− SEM. There was no significant change between the three time points in Gilles de la Tourette syndrome (GTS) patients, whereas healthy controls showed the expected facilitatory effect after PAS_N20_ at T2. Significance levels: *p<.05.

A repeated measures ANOVA (T1, T2, T3) with „group“ as a between-subjects factor (N = 29) revealed a significant interaction between time and group [*F*(2, 54)  = 4.79, *p* = .012]. Post-hoc *t*-tests showed that MEP amplitudes at baseline did not differ between groups [*t*(27)  = −.55, *p* = .587]. MEP amplitudes increased from T1 to T2 [*t*(14)  = −2.41, *p* = .03] and decreased from T2 to T3 in healthy controls [*t*(14)  = 2.44, *p* = .029]. In contrast, there was no mean MEP amplitude difference between T1 and T2 [*t*(13) = 1.07, *p* = .302], or between T2 and T3 [*t*(13)  = −1.35, *p* = .2] in GTS patients (see figure 2). Excluding the 4 patients who were taking medication did not change the results in GTS patients (T1 to T2 [*t*(9)  = 1.16, *p* = .278] and T2 to T3 [*t*(9)  = −0.27, *p* = .79]). T1 and T3 did not differ significantly from each other in either group, indicating that the PAS_N20_ effect had worn off after 30 min (see figure 2).

Concerning the IO curve data, the 3×5 repeated measures ANOVA („time“ x „stimulus intensity”) with “group” as a between-subjects factor did not reveal any significant interactions involving “group”. As expected, there was a significant main effect for stimulus intensity [*F*(1, 26)  = 72.54, *p*<001]. An explorative ANOVA was then conducted for each group separately to see whether there was a detectable effect of PAS_N20_ in one of the groups.

**Figure 3:**
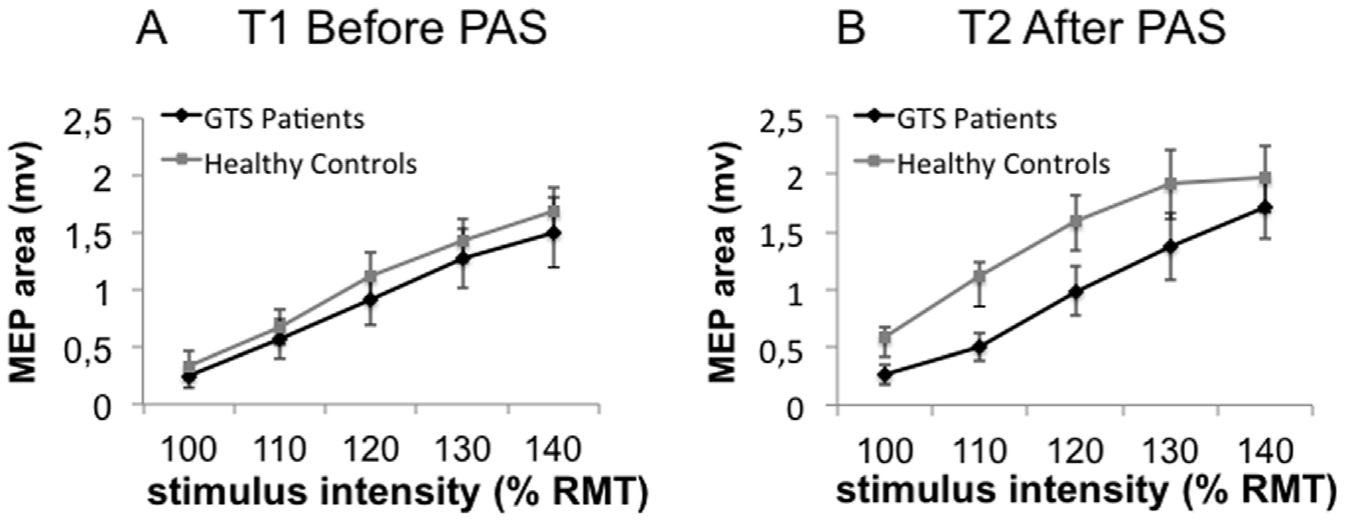
Input-output curves at T1 and T2. Data shown are mean values +/− SEM. **A** Time 1, before PAS_N20_: MEP amplitudes increased significantly in both groups with increasing stimulus intensity. There was no difference between the slopes of the groups at baseline. **B** Time 2, after PAS_N20_: MEP amplitudes of the IO curve increased significantly with increasing stimulus intensity. GTS patients IO curve after PAS did not differ from baseline, while healthy controls showed a general increase in the IO curve after PAS.

The 3×5 repeated measures ANOVA („time“ x „stimulus intensity”) revealed no significant interaction of time and stimulus intensities for IO curve data in controls. As expected, there was a significant main effect of stimulus intensity with higher intensities eliciting higher MEP amplitudes [*F*(1, 56)  = 44.07, *p*<001] and a marginally significant effect for time [*F*(1, 28)  = 3.07, *p* = 06]. Explorative post-hoc t-tests showed that overall mean MEP amplitudes at T2 were significantly larger than those at T1 [*t*(27) = −.5.59, *p* = .02] indicating a general increase in cortico-spinal excitability after PAS_N20_ in the control group, corroborating the results from the main experiment (see figure 3). The same 3×5 repeated measures ANOVA („time“ x „stimulus intensity”) run for the GTS group only yielded significant results for stimulus intensity [*F*(1, 24)  = 22.76, *p*<001] indicating a normal ascending response of MEPs to higher intensities. There was no PAS_N20_ effect in GTS patients (see figure 3).

The amount of MEP change following PAS_N20_ irrespective of the direction of change (|MEP T2-T1|), did not differ between GTS patients and healthy controls [t(27) = −1.04, p = .31], indicating that overall, plasticity effects were comparable. However, more GTS patients than healthy controls (57% compared to 33%) showed an LTD-like change in response to PAS_N20_.

**Figure 4:**
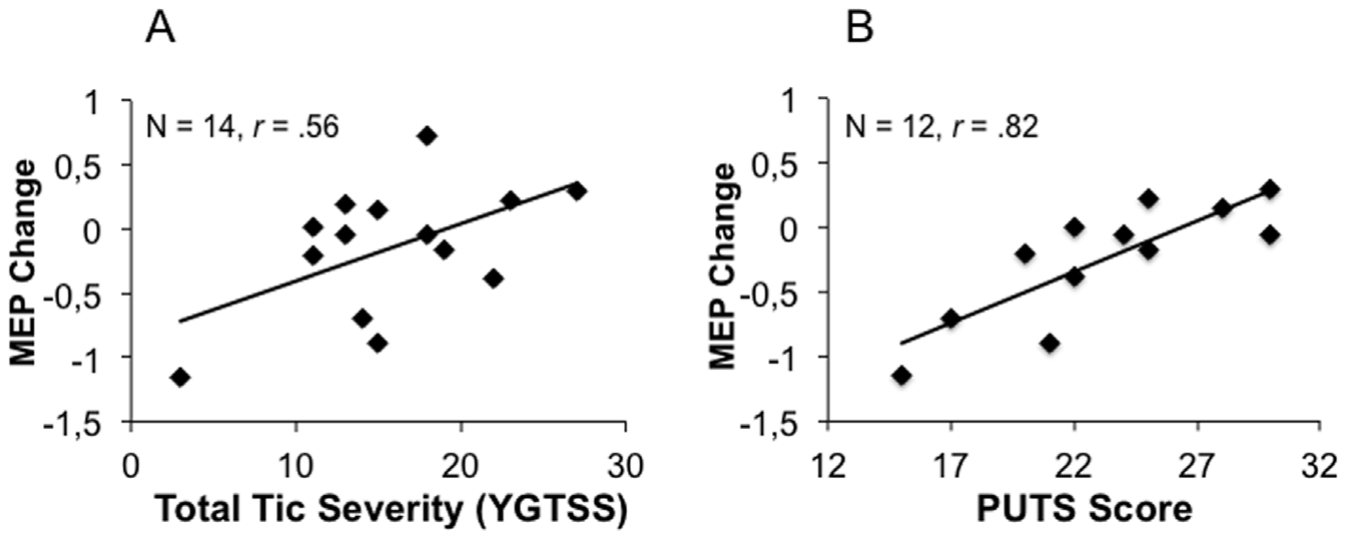
Correlations between induced synaptic plasticity and clinical measures. **A** Correlation between total tic severity (without impairment) as determined with the Yale Global Tic Severity Scale (YGTSS) and change in motor evoked potentials (MEP) from T1 to T2 in patients. **B** Correlation between premonitory urges as determined with the Premonitory Urges for Tics Scale (PUTS) and MEP change from T1 to T2 in patients.

The mean resting motor threshold did not differ between the groups. However, variability was higher in GTS patients (min  = 35; max  = 62) than in healthy controls (min  = 33; max  = 52). YGTSS scores (total tic severity) correlated positively with resting motor threshold (*r* = .56, *p* = .036), i.e. higher tic severity was associated with lower cortical excitability suggesting a generally decreased resting motor excitability in severely affected GTS patients. Moreover, total tic severity correlated positively with MEP change from T1 to T2 (*r* = .56, *p* = .038), indicating LTP-like plasticity in more severely affected patients and LTD-like plasticity changes in patients with fewer tics (see figure 4a). The correlation between MEP change from T1 to T2 and the DCI was not significant (*r* = −.36, *p* = .21). Correlations between the resting motor threshold, and MEP change with clinical measures are reported in table 3. Correlations between MEP change and resting motor threshold with the IO curves are reported in table S1.

**Table 3:** Correlations with Clinical Score.

	MEP Change T2–T1	Resting Motor Threshold
YGTSS Total Tic Frequency	*r* = .53, *p* = .053	*r* = .69, *p* = .007**
YGTSS Total Tic Intensity	*r* = .63, *p* = .016*	*r* = .59, *p* = .027*
YGTSS Total Tic Severity (vocal & motor tic severity)	*r* = .56, *p* = .038*	*r* = .56, *p* = .036*
YGTSS Impairment	*r* = −.08, *p* = .8	*r* = .54, *p* = .047*
Total YGTSS Score	*r* = .25, n.s.	*r* = .67, *p* = .008**
(total tic severity & impairment)		
DCI	*r* = −.36, *p* = .21	*r* = −.05, *p* = .86
PUTS	*r* = .82, *p* = .001**	*r* = .53, *p* = .08

Correlations (*r*) between motor evoked potential (MEP) changes caused by paired associative stimulation (PAS)/resting motor threshold and symptom severity as measured by sub-scales of the Yale Global Tic Severity Scale (YGTSS), the Diagnostic Confidence Index (DCI) (n = 14) and the Premonitory Urge for Tics Scale (n = 12) in Gilles de la Tourette syndrome (GTS) patients.

Significance levels: *p<.05, **p<.01.

Information about premonitory urges was obtained from 12 patients. Premonitory urges and tic severity were positively associated (*r* = .63, *p* = .038). Moreover, the strength of premonitory urges was highly correlated with MEP change (*r* = .82, *p* = .001) indicating that patients with higher LTP-like changes had stronger premonitory urges while patients with stronger LTD-like responses reported fewer or less severe urges (see figure 4b). There was no correlation between MEP change and current intake of medication (*r* = .02, *p* = .96) or previous medication (*r* = −.18, *p* = .61)

### Behavioural Results

**Figure 5:**
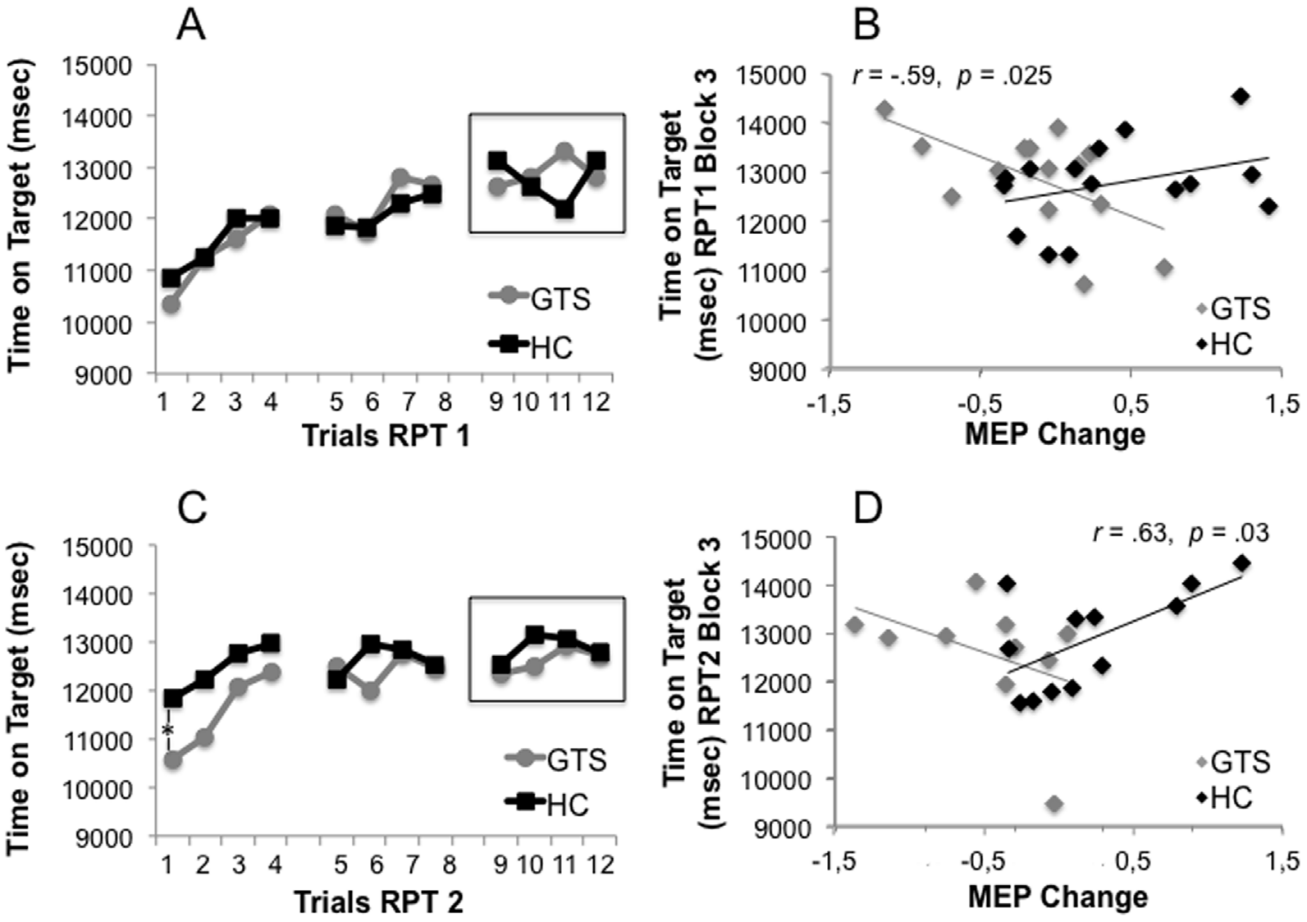
Association between motor skill acquisition and consolidation with synaptic plasticity. **A** Learning curves at time 1, immediately after the paired associative stimulation paradigm (PAS _N20_), of Gilles de la Tourette syndrome (GTS) patients and healthy controls (HC) in the rotary pursuit task across 3 blocks consisting of 4 trials respectively. **B** Mean values of the last block of the learning curves at time 1, immediately after PAS_N20_, correlated significantly negatively with MEP change in GTS patients (n = 14) but not in healthy controls (n = 15). **C** Learning curves of the rotary pursuit task in both groups at time 2, 9 months after PAS_N20_. **D** Mean values of the last block of the learning curves correlated significantly positively with MEP change in healthy controls (n = 12) but not in GTS patients (n = 10).

Patients and healthy controls showed a normal learning curve at time 1 (see figure 5a). A repeated measures ANOVA (“trial” x “time” x “group”) showed a significant linear contrast for learning in both groups at both times during the rotary pursuit task [*F*(1, 20)  = 69,75, *p*<001]. The assumption of sphericity was violated for the within subjects tests, therefore the results of the multivariate tests will be reported. The only significant result apart from the linear learning across trials was a significant three-way interaction between learning curve, time and group [*F*(11, 10)  = 3,67, *p* = 025]. Post hoc t-tests revealed that while there was no difference between patients and healthy controls in the first trial of the rotary pursuit task at time 1 [*t*(27) = .61, *p* = .55], healthy controls started their second learning curve (time 2) at a significantly higher level than GTS patients [*t*(20) = 2.23, *p* = .037] (see figure 5c).

Mean learning in block 1 and 3 at time 1 was significantly negatively correlated with MEP change in GTS patients [*r* = −.65, *p* = .01 for block 1; *r* = −.39, *p* = .17 for block 2; *r* = −.59, *p* = .03 for block 3] (see figure 5b), whereas learning at time 1 was not significantly correlated with amplitude change in healthy controls [*r* = .19, *p* = .49; *r* = .19, *p* = .51; *r* = .36 *p* = .19] (see figure 5b). In healthy controls, there was a relation between the extent of LTP induced at time 1 and motor learning 9 months later. Size of LTP correlated significantly with mean time on target in block 2 and 3 of the rotary pursuit task at time 2 [*r* = .33, *p* = .23; *r* = .58, *p* = .05; *r* = .63, *p* = .03] (see figure 5d). Amplitude change at time 1 and time on target at time 2 were not significantly correlated in any of the blocks in GTS patients [*r* = −.51, *p* = .14; *r* = −.4, *p* = .25; *r* = −.42, *p* = .23] (see figure 5d). The size of the correlations for GTS patients at time 2 is similar to the correlations at time 1. Correlations may have missed significance because the sample may have been underpowered. Therefore, we will report the variance in motor performance (*r^2^*) at time 1 and 2 that can be explained by variance in synaptic plasticity. In healthy controls, 7% of the variance in motor learning in the rotary pursuit task 1 can be explained by PAS_N20_ related synaptic plasticity. In contrast, 31% of the variance in motor learning in the rotary pursuit task 2 can be explained by synaptic plasticity induced 9 months earlier. For GTS patients, 33% of the variance in the rotary pursuit task 1 and 26% of the variance in the rotary pursuit task 2 can be accounted for by changes in synaptic plasticity induced by PAS_N20_. This measure does not reflect causality but merely assesses the size of the association without taking sample size into account. According to these results, there is a difference in the association between motor learning and synaptic plasticity between time 1 and 2 in GTS patients but it is not as clear as in healthy controls.

Performance in the rotary pursuit task 1 and the rotary pursuit task 2 did not correlate significantly with the total YGTSS score or premonitory urges.

## Discussion

The main finding of this study is that M1 synaptic plasticity in adults with uncomplicated GTS differs from healthy controls. As expected, mean MEP amplitudes following PAS_N20_ increased in healthy controls [36], whereas there was no overall change in GTS patients. However, if the absolute MEP amplitude change was taken into account rather than the mean change, synaptic plasticity was not reduced in GTS but bi-directional. More GTS patients than healthy controls showed an LTD-like effect following PAS_N20_ that was correlated with less severe urges and fewer tics. Both groups performed equally well in the motor task immediately following PAS_N20_. However, healthy controls performed significantly better than GTS patients in the first trial of the motor task 9 months later, indicating that long-term consolidation processes differed between the two groups.

Two studies, using iTBS and HFS, have previously shown reduced synaptic plasticity in GTS patients with comorbidities compared to healthy controls [6], [7]. Our results confirm those findings in patients with uncomplicated GTS and extend them by showing that plasticity is not reduced on the individual level but that the majority of patients show LTD-like plasticity in response to PAS_N20_. Wu et al. (2012) also reported “substantial variability” in their GTS sample using iTBS to induce LTP-like plasticity, but did not report whether absolute changes in amplitude were similar in GTS patients and healthy controls [6].

The main question addressed in the present study was whether synaptic plasticity can be related to impaired motor learning in GTS patients. Our data suggests that aberrant synaptic plasticity in GTS is related to reduced long-term consolidation of motor skills in the rotary pursuit task. Rajji and colleagues (2011) found that TMS induced LTP did not enhance performance in the rotary pursuit task 45 min after PAS_25_ but that it enhanced motor learning one week later [33]. Synaptic plasticity can be divided into short-term effects, lasting from a couple of minutes up to hours, and long-term effects, lasting from hours to months [47]. Short-term LTP is likely achieved by a modification in the likelihood of transmitter release, while long-term LTP might be related to more persistent postsynaptic, structural changes [47]. Based on the established biological mechanisms, Rajji and colleagues (2011) proposed that long-term improvements in the rotary pursuit task, beyond practise effects, might be achieved by PAS_25_-induced long-term structural changes in M1. The present study confirms this finding by showing that synaptic plasticity was unrelated to motor learning 45 min after PAS_N20_ but correlated positively with motor learning in healthy controls 9 months after PAS_N20_. The results indicate a long-term beneficial effect of induced LTP-like plasticity in healthy controls. However, GTS patients did not express LTP-like changes in response to PAS_N20_.

Although motor skill acquisition in the rotary pursuit task was normal in GTS patients [48], they started their learning curve at a significantly lower level than the control group in the second motor learning session, 9 months after PAS_N20_. In other words, long-term consolidation of motor learning appeared to be stronger in healthy controls than in GTS patients. These results could be accounted for in two different ways. Either long-term consolidation of motor learning in GTS patients is impaired in general, or GTS patients did not benefit from PAS_N20_ because no LTP was induced. However, if long-term consolidation of motor learning is generally impaired in GTS patients, this may also be related to aberrant synaptic plasticity. TMS-induced LTP is thought to rely on the same biological processes as learning experiences in a natural environment [14]. If GTS patients show LTD-like plasticity instead of LTP-like plasticity in response to PAS_N20,_ they may also differ with respect to biological processes in motor learning tasks. This would be an interesting question to address in an independent experiment.

Suppa and colleagues (2011) and Wu and colleagues (2012) have pointed out that synaptic plasticity in GTS patients may be altered because of meta-plasticity effects, which may occur as a consequence of tics [6], [7]. The excitability of a neuron depends, in part, on its firing history [49]–[51]. If a neuron has been highly active, self-regulatory feedback mechanisms can scale the excitability of the neuron down [49]. Although this assumption has been voiced twice, there is no detailed account of how meta-plasticity may affect synaptic plasticity in GTS patients. We would like to discuss a theoretical framework that could account for our results. However, it should be clearly stated that all results in the present study are based on correlational analyses and cannot provide any information about causality.

According to the Bienenstock-Cooper-Munro rule, there is a floating threshold, which determines the amount of activity needed to elicit LTP or LTD. The activation needed is a function of the average postsynaptic activity levels [52], i.e. neurons that have been relatively over-active are more likely to decrease their synaptic weight. Although it is difficult to apply these homeostatic effects found in single cells to a complex system that develops over many years, it can be speculated that GTS brains that have adapted to an over-active striatal system, may react differently to PAS_N20_ stimulation than do healthy brains.

If there is a long-term compensatory mechanism, it might not be as simple as single cell threshold adaptation though. Most GTS patients gain increased control over their symptoms during adolescence [53], thus compensatory effects may be associated with the development of the prefrontal cortex. This hypothesis is supported by several studies showing that tic severity was associated with enhanced cognitive control and structural changes in the prefrontal cortex [54], [55]. The prefrontal cortex might not exert inhibitory control but may serve to bias response competition in motor areas [56], [57]. Based on this assumption Jeyong et al. (2013) have proposed that the prefrontal cortex may be hyperactive in GTS patients and that this hyperactivity may be compensated for in adolescence by structural and functional changes in the long-range neural pathways that link the prefrontal cortex to those motor areas [58]. Another result of those compensatory mechanisms could be an overall change in synaptic weights in M1, thereby creating a bias towards LTD-like plasticity in response to excitatory stimulation. However, the assumptions described should be addressed in a longitudinal study.

Several findings in the present study would support the assumption of a compensatory mechanism. Remarkably, LTD-like changes were strongly associated with fewer premonitory urges and fewer tics. Fewer tics were in turn associated with fewer premonitory urges. Moreover, those GTS patients who reacted with LTD-like plasticity instead of LTP-like plasticity were better at motor skill acquisition in the rotary pursuit task. If LTD-like plasticity were indeed a compensatory mechanism, then these results would indicate that patients who compensate more successfully for their tics and urges may also develop strategies in dealing with motor learning more successfully.

An alternative explanation for LTD-like plasticity in GTS would have been an increased cortical excitability at baseline reflecting a homeostatic reaction of an „overexcited“ brain. Reversed effects of TMS protocols that normally induce LTP, such as PAS, occur in healthy volunteers if cortico-spinal excitability is altered at baseline and have been attributed to homeostatic meta-plasticity [59], [60]. However, in this study there was no correlation between resting motor threshold and MEP change, suggesting no direct association between LTD-like changes and heightened baseline cortical excitability. On the contrary, correlations between resting motor threshold and tic severity showed that more severely affected patients had lower cortical excitability at baseline than less severely affected patients, although in keeping with previous studies, mean resting motor threshold at baseline did not differ between GTS and healthy controls [61], [62]. To our knowledge, this is the first study to show an association between resting motor threshold and tic severity measured by the YGTSS.

Another finding in this study was that IO curves did not differ between the groups at baseline, which is an interesting result with respect to previous inconsistent IO findings in GTS. While one study found a shallower slope in GTS patients as compared to healthy participants, suggesting reduced cortico-spinal excitability at rest [63], another study could not replicate this difference at rest but found shallower IO slopes in GTS patients during movement preparation [64]. However, tic severity was much higher in the sample investigated by Orth and et al. (2008) than in the study by Heise et al. (2010) hence discrepancies between studies may be attributable to clinical differences in the populations investigated, such as tic severity or efforts to control tics [65]. Our finding that tic severity was positively correlated with resting motor threshold, i.e. reduced excitability at rest, corroborates the assumption that tic severity might be associated with cortical excitability. However, it remains unclear whether this is a short-term effect, possibly due to the necessity to control tics for the duration of the experiment.

The main limitation of this study is its small sample size. The population investigated was very homogeneous though, making it more likely that the results can be generalized to other uncomplicated GTS patients despite the sample size. However, approximately 90% of GTS patients suffer from comorbidities [66], hence the findings reported in this study might not be valid for the whole population of GTS patients. Further limitations of this study include the possibility that past and present intake of medication may have influenced the results. However, controlling for present intake of medication did not alter the results and neither present nor past intake of medication correlated with MEP change. Also, right hand or finger tics during MEP measurements could have influenced PAS_N20_ responses but this is unlikely because no tics occurred during the assessments of MEPs and IO curves. We cannot exclude the possibility though that ticcing or tic suppression at other times during the experimental procedure influenced results. Furthermore, we cannot deduce whether the difference in long-term consolidation between the groups is a general problem in GTS or whether it was due to the absence of induced LTP at time 1. Further research will be needed to determine whether motor skill consolidation is generally impaired in GTS patients. Moreover, it would be interesting to induce synaptic plasticity in children with GTS in order to investigate whether they show a normal response to PAS. If our findings of aberrant plasticity in GTS reflect a compensatory mechanism, it should not be present before puberty.

## Conclusions

Synaptic plasticity in response to PAS_N20_ differed between a small sample of GTS patients and healthy controls. The majority of patients responded with LTD-like changes, while the majority of healthy participants responded with LTP-like changes. Patients also showed reduced motor skill consolidation as compared to healthy controls 9 months after PAS_N20_. Overall, patients may not benefit from the long-term structural changes that occur when LTP-like effects are induced. Although LTP was artificially induced in this study these results may help to explain impairments in cortex-based motor learning in GTS patients more generally. Moreover, GTS patients with strong premonitory urges and more severe tics tended to show physiological LTP-like plasticity. In contrast, less severely affected patients had LTD-like responses, suggesting a compensatory mechanism.

## Supporting Information

Table S1 **Correlations between physiological measures.** Correlations between the input-output (IO) slopes before the paired associative stimulation (PAS; time 1), immediately after PAS (time 2) and 30 min. after PAS (time 3) with motor evoked potential (MEP) changes from time 1 to time 2 and the resting motor threshold in Gilles de la Tourette (GTS) patients and healthy controls. Significance levels: *p<.05.
(DOCX)
