# Supplementary material for: Altered Synaptic Plasticity in Tourette's Syndrome and Its Relationship to Motor Skill Learning
Source: PLoS One. 2014 May 30;9(5):e98417. doi: 10.1371/journal.pone.0098417 (PMC4039486; doi:10.1371/journal.pone.0098417)
Supplement: Table S1 — Correlations between physiological measures. Correlations between the input-output (IO) slopes before the paired associative stimulation (PAS; time 1), immediately after PAS (time 2) and 30 min. after PAS (time 3) with motor evoked potential (MEP) changes from time 1 to time 2 and the resting motor threshold in Gilles de la Tourette (GTS) patients and healthy controls. Significance levels: *p<.05. (DOCX) [file pone.0098417.s001.docx]

| **Table S1:** *Correlations between physiological measures* | | | | |
| --- | --- | --- | --- | --- |
|  | MEP Change T2 – T1 | | Resting Motor Threshold | |
|  | GTS | Healthy | GTS | Healthy |
| IO Slope 1 | *r* = -.45, *p* = .12 | *r* = .16, *p* = .58 | *r* = -.68, *p* = .011* | *r* = .11, *p* = .7 |
| IO Slope 2 | *r* = -.2, *p* = .52 | *r* = .21, *p* = .46 | *r* = -.68, *p* = .011* | *r* = -.23, *p* = .41 |
| IO Slope 3 | *r* = -.19, *p* = .53 | *r* = .2, *p* = .48 | *r* = -.68, *p* = .011* | *r* = .19, *p* = .49 |
